# Air Ambulance: Antimicrobial Power of Bacterial Volatiles

**DOI:** 10.3390/antibiotics11010109

**Published:** 2022-01-14

**Authors:** Alexander Lammers, Michael Lalk, Paolina Garbeva

**Affiliations:** 1 Department of Cellular Biochemistry and Metabolomics, University of Greifswald, 17487 Greifswald, Germany; lalk@uni-greifswald.de; 2 Department of Microbial Ecology, Netherlands Institute of Ecology (NIOO-KNAW), 6708 PB Wageningen, The Netherlands

**Keywords:** volatile organic compounds, volatiles, chemical ecology, metabolomics, antibacterial, antifungal, antibiotics, antimicrobial resistance crisis

## Abstract

We are currently facing an antimicrobial resistance crisis, which means that a lot of bacterial pathogens have developed resistance to common antibiotics. Hence, novel and innovative solutions are urgently needed to combat resistant human pathogens. A new source of antimicrobial compounds could be bacterial volatiles. Volatiles are ubiquitous produced, chemically divers and playing essential roles in intra- and interspecies interactions like communication and antimicrobial defense. In the last years, an increasing number of studies showed bioactivities of bacterial volatiles, including antibacterial, antifungal and anti-oomycete activities, indicating bacterial volatiles as an exciting source for novel antimicrobial compounds. In this review we introduce the chemical diversity of bacterial volatiles, their antimicrobial activities and methods for testing this activity. Concluding, we discuss the possibility of using antimicrobial volatiles to antagonize the antimicrobial resistance crisis.

## 1. Antimicrobial Resistance: A Global Crisis

Many would argue that the discovery of antibiotics changed the world of medicine. Penicillin is often reported as the first antibiotic available to the public, whereas it was actually sulfamidochrysoidine [1,2]. In contrast to penicillin, sulfamidochrysoidine, which was sold under the trade name Prontosil, was toxic for humans and disappeared quickly from the market and history books [1]. However, with the introduction of penicillin in the 1940s, antibiotics have saved millions of lives and the subsequent years are often referred as the “golden age” of antibiotics due to the discovery of numerous novel classes [3]. In fact, in the end of the 1960s around 24 novel classes of antibiotics were introduced to the market [2], but since the 1970s only three classes, namely pseudomonic acids [4,5], oxazolidiones [6] and lipopeptides [7] have been introduced to the market. One of the latest promising antibiotics is the peptide teixobactin, which due to its highly conserved targets is unlikely to induce resistance but is still not available on the market [8]. Interestingly, Alexander Fleming understood the fragility of the powerful tool antibiotic and warned at his Nobel Prize speech in 1945: “The time may come when penicillin can be bought by anyone in the shops. Then, there is the danger that the ignorant man may easily underdose himself and by exposing his microbes to non-lethal quantities of the drug make them resistant” [9]. In his speech, Fleming indicated the risk of antimicrobial resistance—an issue that we are facing today.

How is it possible that, despite Alexander Fleming’s warning, resistant pathogens could become a global issue? In human medicine antibiotics are often overused as well as misused. That means that antibiotics are prescribed as a prophylactic or the actual pathogen is not identified before prescription. Furthermore, patients may take antibiotics without referring to a doctor. Additionally, the massive and prophylactic use of antibiotics in agriculture causes resistance which may be transferred to humans. Globalization makes the spread of resistant pathogens very easy and the hurdles for (economically driven) pharmaceutical companies are very high. However, these are only the main reasons and have already been reviewed in detail [10]. This misuse of antibiotics has led to the development of numerous antibiotic-resistant pathogens resulting in an antimicrobial resistance crisis on a global scale [10,11,12]. There are already at least 700,000 deaths caused every year by drug-resistant pathogens globally [13] and scientists predict that by 2050 antibiotic resistance could be responsible for over 10,000,000 deaths per year [14]. Moreover, the global COVID-19 pandemic has caused an increased use of antibiotics due to bacterial co-infections or prophylactic treatment with antibiotics to avoid those co-infections [15]. In order to address this crisis, we need to explore alternatives to classical antibiotics. Numerous pharmaceutical options to counter the antimicrobial resistance crisis are under discussion [16,17]. One approach is to discover novel sources for antimicrobial compounds that can be developed into future treatments. In recent years, more and more studies have reported volatiles with antimicrobial activity which indicates that volatiles might play an important role in countering the antimicrobial resistance crisis [18,19,20,21]. For example, the new volatile antibiotics albaflavenone and pentalenolactone produced by *Streptomyces coelicolor* and *Streptomyces avermitilis*, respectively were discovered [22].

In this review we introduce the chemical characteristics of volatiles, provide an overview of antimicrobial volatiles of bacterial origin and the main methods used for testing their antimicrobial activity. Concluding, we discuss the potential role of volatiles as a novel class of antimicrobials.

## 2. Biochemistry of Volatiles: Diverse and Diffusive

Volatiles form a chemical class of molecules that all have one characteristic in common: their high vapor pressure at ambient temperatures [23]. Additionally, volatiles are characterized by their low molecular weights of a maximum of 200–500 Dalton, low boiling points and often lipophilic moieties [23,24,25]. Volatiles significantly differ from soluble compounds in one key characteristic: they do not depend on solvents. Although many volatiles are nonpolar showing low solubility in water due to their restricted number of functional groups, this solubility is sufficient to allow dissemination into the water phase. Hence, volatiles spread fast in both the gas and water phase [26]. Via the gaseous phase, volatiles can spread in highly complex ecosystems such as soil [27], insect nests [28] and spider nests [29] and can fulfill functions such as communication [30] and antimicrobial defense [31] which cannot be performed by solvents due to the lack of effective spreading.

**Figure 1:**
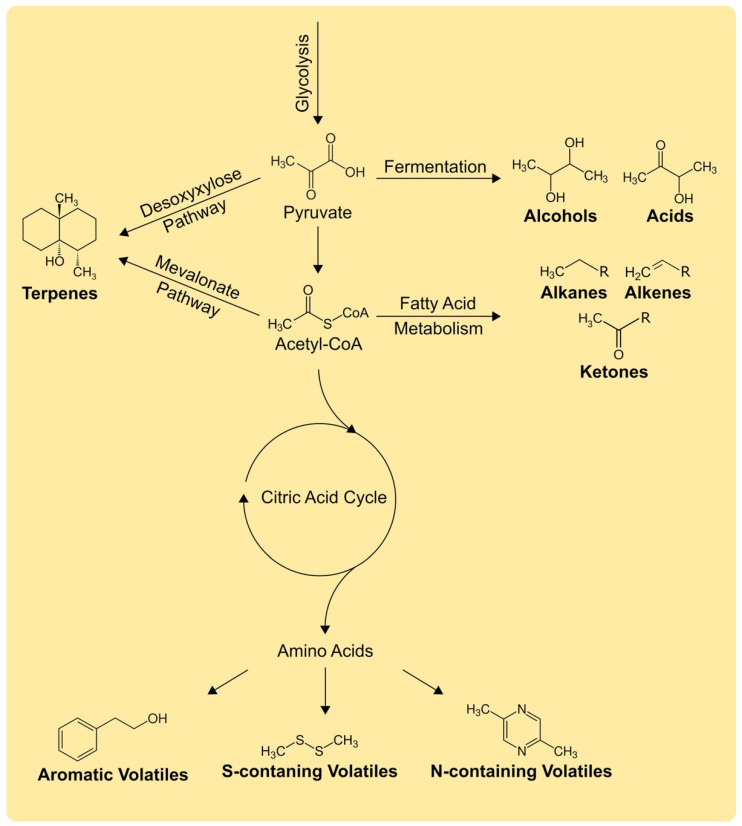
Overview of main biochemical pathways for the production of bacterial volatiles. The chemical structures show representative examples: alcohols (2,3-butanediol), acids (acetoin), alkanes (general structure), alkenes (general structure), ketones (general structure), terpenes (geosmin), aromatic volatiles (2-phenylethanol), S-containing volatiles (dimethyl disulfide) and N-containing volatiles (2,5-dimethylpyrazine). Details are described in the main text.

Volatiles belong to diverse chemical classes such as hydrocarbons, aromates, alcohols, aldehydes, acids, esters, amines and thiols [23]. Bacterial volatiles in particular were for the first time systemically reviewed by Schulz and Dickschat in 2007 describing in detail the biosynthesis of common volatiles like fatty acids or sulfur-compounds which are produced by most bacteria but also rare volatiles like halogenated compounds [32]. Volatile biosynthesis is usually based on pyruvate and therefore takes place in primary metabolism (Figure 1) [24,33]. Pyruvate can be directly metabolized to short acids or alcohols [33]. *Bacillus* spp. for example, which are well known for producing volatiles as we will discuss later in this review, produces mainly 2,3-butanediol and acetoin via fermentation [34].

Under aerobic conditions pyruvate will be metabolized to acetyl-CoA and can enter the fatty acid anabolism, citric acid cycle or be converted to terpenes. The fatty acid metabolism results mainly in alkanes, alkenes, aliphatic alcohols and ketones. The β-oxidation with acetyl-CoA results only in fatty acids with even numbers, for odd-chain fatty acids propionyl-CoA replaces one acetyl-CoA in the final step [33]. Typical volatile products from the fatty acid pathway are aldehydes such as nonanal, ketones such as nonan-2-one or fatty acids such as nonanoic acid [26].

When acetyl-CoA enters the citric acid cycle it is metabolized to the precursors of most amino acids, which act again as precursors for aromatic, nitrogen-containing and sulfur-containing volatiles [24]. Aromatic volatiles are metabolized based on aromatic amino acids or directly via the shikimate pathway. 2-Phenylethanol for example, a common aromatic volatile compound, can be metabolized based on the amino acid phenylalanine [32,33]. Pyrazines are usually based on amino acids due to their nitrogen-containing aromatic ring. Additionally, sulfur-containing volatiles such as dimethyl disulfide and dimethyl trisulfide which are produced by most bacteria are based on methionine [32].

Another important group of volatiles are terpenes, which are well known to be present in essential oils [35] but in recent years have also been discovered frequently in bacterial volatile blends [36]. Terpenes are synthesized via the mevalonate or desoxyxylulose pathway [26]. The mevalonate pathway starts with acetyl-CoA from the glycolysis and was for a long time assumed to be the only way to biosynthesize isopentenyl diphosphate and dimethylallyl diphosphate, the precursors of terpenes. However, the desoxyxylulose pathway starts with pyruvate, the precursor of acetyl-CoA [37,38]. The sesquiterpene geosmin, which is produced by actinomycetes, myxobacteria and cyanobacteria has a characteristic soil-like smell [32,33]. Interestingly, different geosmin synthases were found in actinobacteria compared to myxobacteria and cyanobacteria [36].

Alongside organic volatiles, bacteria produce inorganic volatiles such as hydrogen sulfide, hydrogen cyanide, nitric oxide or ammonia [36]. Ammonia for example is produced in high amounts by *Streptomyces* spp. and is produced within amino acid catabolism [39,40]. Moreover, ammonia was shown to be antimicrobial against Gram-positive and -negative bacteria and can act therefore as a long-distance (several centimeters) antibiotic [39].

## 3. Bacterial Antimicrobial Volatiles: An Overview

Recently, an increasing number of studies have revealed individual volatiles or volatile blends of bacterial origin with antimicrobial activities [18]. These studies indicate that the antimicrobial potential of volatiles is as diverse as their biochemistry (Table 1). A bulk of the investigated volatiles were reported for their antifungal activities and cause for reduced hyphal extension and/or hyphal biomass as well as spore germination. For example, the volatile blends produced by *Paenibacillus polymyxa* Sb3-1 and *Bacillus velezensis* I3 were shown for their antifungal activities [41,42]. However, several studies also reported bacteria that produce antibacterial [43] or anti-oomycete volatiles [44]. Beyond that, some volatiles were reported for their broad antimicrobial spectrum. For example, the volatile 2,5-*bis*(1-methylethyl)-pyrazine produced by *Paenibacillus* sp. AD87 revealed a broad-spectrum activity against a range of human and plant pathogens. The volatile inhibited the bacterial pathogens *Escherichia coli* and *Staphylococcus aureus*, the fungal pathogens *Fusarium culmorum* and *Rhizoctonia solani* as well as the yeast *Candida albicans* [21,45]. At the same time 2,5-*bis*(1-methylethyl)-pyrazine showed very low toxicity on mammalian cells [45]. Another example of volatiles with broad spectrum antimicrobial activity is γ-Butyrolactones, active against fungi, yeasts, and bacteria [46].

### 3.1. Sulfur-Containing Volatiles

Often, described antimicrobial volatiles of bacterial origin are alcohols, pyrazines and sulfides (Table 1). Especially sulfur-containing volatiles such as dimethyl sulfide, dimethyl disulfide and dimethyl trisulfide are often reported because they are commonly produced by bacteria and apparently have strong antimicrobial activities [26]. For example, dimethyl disulfide has antibacterial potential, as it revealed bacteriostatic effects against the two plant pathogens *Agrobacterium tumefaciens* and *Agrobacterium vitis* [74]. However, the volatile is also known for its antifungal activity. Currently, dimethyl disulfide is used as a novel fumigant (PALADIN^®^) to target soil-borne plant pathogens [25]. The chemically related volatile dimethyl trisulfide significantly inhibited the growth of three human pathogens *Serratia marcescens, Escherichia coli* and *Staphylococcus aureus* [75]. As well as linear sulfur-containing compounds, scientists also report from aromatic sulfur-containing compounds. Gotor-Vila reported thiophene in the volatilome of *Bacillus amyloliquefaciens* CPA-8 and showed its antifungal activity [47].

### 3.2. Bacillus and Streptomyces as Volatile-Producers

Most likely, all bacterial genera produce volatiles, but *Bacillus* species especially are often reported to produce volatiles with antimicrobial potential (Table 1). For example, volatiles emitted by *Bacillus amyloliquefaciens* FZB42 including benzaldehyde, 1,2-benzisothiazol-3(2*H*)-one and 1,3-butadiene showed strong inhibitory activities against *Ralstonia solanacearum*, a bacterial plant pathogen causing wilt disease [49]. Other studies report the same from *Bacillus amyloliquefaciens* strains but with antifungal activities [47,48,50]. Alongside *Bacillus*, *Streptomyces* species were also often investigated because the genus is well known for its antimicrobial potential, including volatiles [39,76]. For example, terpenoid volatiles are abundantly emitted by *Streptomyces* species and pose interesting antimicrobial properties. The soil isolate, *Streptomyces albidoflavus*, was shown to produce a sesquiterpene, namely albaflavenone, with antibacterial properties [77]. Lately, albaflavenone was isolated from other *Streptomyces* species and fungi [78,79]. Another sesquiterpene compound with antibacterial activity is dihydro-β-agarofuran, produced by *Streptomyces* species [80]. The antimicrobial volatile pentalenolactone emitted by *Streptomyces roseogriseus* was discovered to possess antibacterial activity against Gram-positive and Gram-negative bacteria. Furthermore, anisole, emitted by *Streptomyces albulus*, was reported to inhibit the growth of fungal plant pathogens *Sclerotinia sclerotiorum* and *Fusarium oxysporum* [81].

### 3.3. Co-Cultivation and Volatile Blends

Some studies revealed that the co-cultivation of different microbial strains can influence the metabolism of bacteria (Table 2). For example, *Paenibacillus* sp. AD87 was shown to produce the antimicrobial volatile 2,5-*bis*(1-methylethyl)-pyrazine when cultivated alone. After co-cultivation of *Paenibacillus* sp. AD87 together with the phylogenetically different strain *Burkholderia* sp. AD24, the headspace concentration of 2,5-*bis*(1-methylethyl)-pyrazine was increased [21]. Due to the growth inhibiting effect of 2,5-*bis*(1-methylethyl)-pyrazine on *Burkholderia* sp. AD24, it is likely that the volatile production of *Paenibacillus* sp. AD87 was increased as a response to the competition. As well as the increased production of the pyrazine, the co-cultivation resulted in a changed gene expression in both bacteria. For example, *Burkholderia* sp. AD24 showed an increased expression of a type IV secretion system gene which is involved in virulence. *Paenibacillus* sp. AD87 showed increased expression of genes involved in antibiotic resistance. Another study cultivated five bacterial strains together, among others likewise *Paenibacillus* sp. AD87 and *Burkholderia* sp. AD24 [82]. The analysis of the collective volatile blend of the five bacteria revealed among others 2,5-*bis*(1-methylethyl)-pyrazine as well. Interestingly, the pyrazine was only found in the collective volatile blend but neither in the volatilome of *Paenibacillus* sp. AD87 nor in one of the others indicating production activation by the co-cultivation. Rybakova et al. co-cultivated the bacterium *Paenibacillus polymyxa* Sb3-1 with the fungus *Verticillium longisporum* EVL43 resulting in several up- and downregulations of the volatile blends of both strains, including volatiles that are most likely related to antimicrobial defense [42]. Furthermore, the corporate production of volatiles was shown under lab conditions [83]. By co-cultivation of *Serratia plymuthica* 4Rx13 and *Staphylococcus delphini* without physical contact they produced corporately the volatiles schleiferon A and B. Separately, none of the bacteria was able to produce those products. Furthermore, Abis et al. analyzed the relation between microbial diversity and volatile emission in general [84]. Interestingly, they could show that a reduced microbial diversity in soil correlates with an increased volatile emission and a smaller number of released volatiles. They discussed that these findings might be caused by a bacterial volatile absorption. However, it is certain that the microbial diversity and community influence the volatile blend in an ecosystem, even when the detailed relations still remaining unknown.

Mixtures of volatiles may result in increased antimicrobial activities compared to single volatiles. For example, a mix of four monoterpenes (γ-terpinene, 1S-α-pinene, β-pinene and β-myrcene) revealed strong antibacterial activity against the pathogenic bacteria *Escherichia coli* and *Staphylococcus aureus* [85]. However, as single compounds they revealed little or no antimicrobial activity. Furthermore, for a number of fungal and bacterial isolates, antimicrobial activities of their volatile blend are reported, but the compounds responsible for this activity remained unknown (Table 1). It is plausible that not a single volatile but a mix of compounds is responsible for this activity.

### 3.4. Modes of Action and Abiotic Factors

Although, for many microbial volatiles, powerful antimicrobial activities have been reported, little is known about modes of action of these molecules on the target organisms. Some microbial volatiles can interfere with well-known bacterial chemical communication systems like N-acylhomoserine lactones’ (AHLs) quorum-sensing. Bacteria use AHLs’ quorum sensing to regulate certain phenotype expressions, such as biofilm formation, virulence factor expression, motility and others. Many lactones (10-methylundec-2-en-4-olide, 10-methylundec-2-en-3-olide, 10-methyldodecan-4-olide, 10-methyldodecan-5-olide, others) positively or negatively influenced the quorum-sensing bacterial communication. This influence could be due to the structural similarity between lactones and AHLs. However, other classes of volatiles such as dimethyl disulfide could also impact bacterial quorum sensing communication by significantly suppressing the transcription of AHLs synthase genes [86]. The above discussed 2,5-*bis*(1-methylethyl)-pyrazine resulted in more direct damages which depend interestingly on the concentration. At high levels, the volatile resulted in DNA damage, whereas at low levels cell-wall damages were observed [45]. Likewise, another study investigating the volatile blend activity of a *Streptomyces* species against the oomycete plant pathogen *Peronophythora litchi* showed, among others, cell-wall damages [70]. A study investigating the antimicrobial activity of the monoterpenes linalyl acetate, menthol and thymol indicates that those volatiles modify the membrane permeability which causes leakage of intracellular material [87]. Furthermore, it is likely that the altered membrane allows the volatiles to enter the cells and might cause further damages. Nevertheless, detailed information about the modes of actions of antimicrobial volatiles are lacking yet and need further investigation. Moreover, detailed information about volatile concentrations are mostly lacking which makes clear statements about the ecological relevance of often challenging volatiles [88].

Additionally, the emission of microbial volatiles is influenced by various abiotic factors such as nutrient availability, temperature and pH. For example, a nutrient-poor growing condition triggered higher levels of terpene emission at an early growth stage of the fungal isolate *Fusarium culmorum* [82]. It is plausible that such compounds have strong antimicrobial activity and are important for the producing organisms to survive under competitive interactions. Another study showed changes in antifungal activity of the volatile blend of *Bacillus amyloliquefaciens* CPA-8 when cultivated on different media [47]. Similarly, the production of antifungal volatiles by the mycophagous soil bacterium *Collimonas* was strongly influenced by different nutrient conditions [89].

## 4. Antimicrobial Activity of Volatiles: The Testing Methods

**Figure 2:**
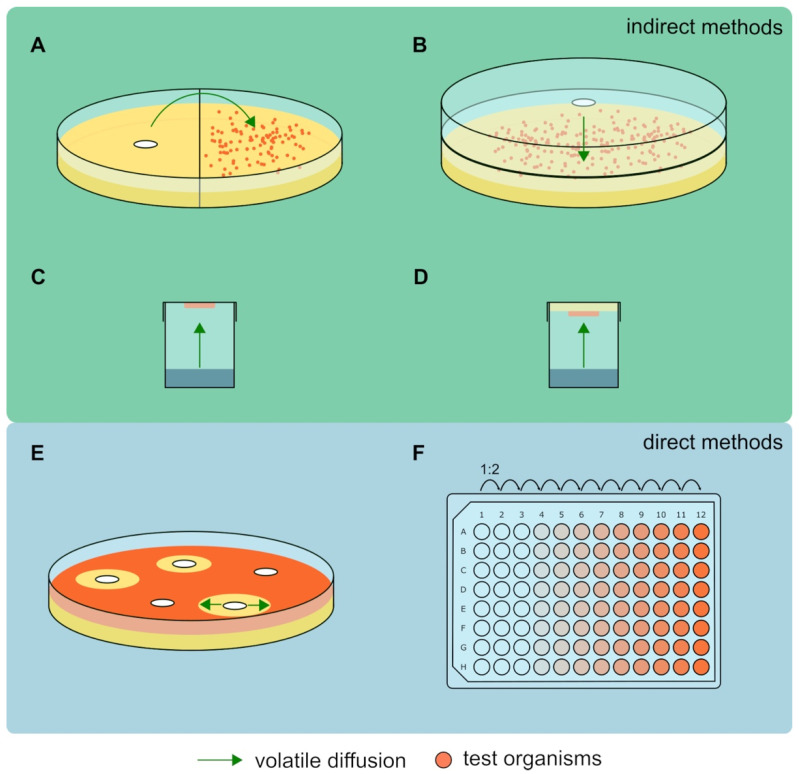
Overview of indirect (**A**–**D**) and direct (**E**,**F**) approaches to test the antimicrobial activity of volatiles. Indirect approaches such as the two-chamber Petri dish (**A**), double plate (**B**), vial (**C**) and AntiBio Vol approach (**D**) compel the volatiles to diffuse through the gas phase to reach the test organisms. In contrast, direct approaches such as the agar diffusion approach (**E**) and the minimal inhibitory test (**F**) allow direct contact between the volatiles and test organisms. Details are described in the main text.

The approaches to test the antimicrobial activity of volatiles can be divided into two main categories: indirect and direct (Figure 2). In indirect approaches the volatiles need to diffuse through the gas phase (usually air) to influence the test organisms. In contrast, direct approaches allow the volatiles to make contact with the test organisms via the liquid phase (usually water) and do not need to diffuse through the gas phase.

One of the most common indirect approaches is the use of two-chamber Petri dishes (Figure 2A) [27,75]. Two-chamber Petri dishes contain a physical wall to divide the inside space into two chambers, making it possible to fill each side with a different media. One side can be inoculated with volatile producing organism or pure volatiles, whereas the other side is inoculated with test organism. After a common incubation, the growth of the test organism is analyzed, e.g., by counting colony forming units or analyzing the growth area. The Petri dishes are commercially available with two, three, or four chambers allowing combination of several organisms or pure compounds with each other. In contrast, the double plate approach is based on standard Petri dishes (Figure 2B) [30]. The bottom and top parts can be filled with different media, whereas one part is inoculated with the volatile producing organisms or pure volatiles and the other part with test organisms. The growth of the test organisms under exposure of the volatiles can be analyzed similarly to the two-chamber Petri dish approach. The vial approach is based on vials filled with solutions containing volatiles (Figure 2C) [90]. The lid contains filter paper with a defined inoculum of test organisms and is exposed to the volatiles before it is incubated in liquid medium to analyze the growth of the test organism. Recently, the AntiBio Vol approach was published (Figure 2D) [91]. Defined biofilms in a 24-well plate were placed upside-down on a second 24-well plate filled with solutions containing volatiles. After common incubation the biofilm is transferred to fresh, liquid broth, incubated shortly and the biomass is analyzed.

In contrast to indirect approaches, direct approaches do not compel the volatiles to actually diffuse through the gas phase, which comes with the advantage of better control of the volatile concentrations but the drawback that those approaches are not suitable with many volatiles due to their frequent lipophilic moiety, which may require the use of organic solvents [23]. The agar diffusion test is widely used to test the antimicrobial activity of pure compounds on solid media (Figure 2E) [46,92]. Cotton discs prepared with pure compounds are placed on agar plates that were inoculated with test organisms. The test compounds diffuse through the agar and may result in a zone of inhibition (ZOI) around the cotton disc. The application of different concentrations of test compounds can be used to determine the minimal inhibitory concentration (MIC). However, a more common approach to investigate the MIC is the two-fold dilution approach which is based on liquid media (Figure 2F). For the approach a defined concentration of the test compound in liquid broth is prepared and subsequently several times 1:2 diluted [93]. Often, this approach is performed using 96-well plates to reach high throughputs with little material usage.

## 5. Concluding Remarks and Future Perspectives

Recent studies clearly demonstrate the ability of various bacteria to produce antimicrobial volatiles that inhibited the growth of either human or plant pathogens, indicating their antibiotic potential and possible application in agriculture and medicine. The demand for new approaches and compounds is high in both agriculture (due to an EU ban of many chemical pesticides) and healthcare (due to antibiotic resistance and side-effects). As the research of antimicrobial volatiles is a newly developing field, there are still many novel volatile compounds to be discovered and chemically characterized. Individual compounds might be commonly found in many often unrelated strains, while others are restricted only to a certain group of strains. Usually, mixtures of compounds are released with widely varying concentrations. Yet, their effects on other organisms and their biosynthesis need to be investigated in more detail in the future. Furthermore, the effects of antimicrobial volatiles need to be evaluated on non-target beneficial (micro)organisms. Many fundamental questions about the modes of action of antimicrobial volatiles and possible resistances remain unanswered and need to be investigated in order to advance our basic knowledge in this research field.

Alongside the treatment of pathogens with volatiles alone, another approach is to combine volatiles with common antibiotics. For example, when exposed to the volatile blend of a *Streptomyces* species, *Bacillus subtilis* showed increased sensitivity against several antibiotics [39]. Interestingly, other studies indicate opposite effects. *Bacillus subtilis* exposed to triethylamine showed reduced sensitivity to tetracycline [94]. However, a selective combination of volatiles and common antibiotics may be a successful tool against resistant bacteria in future.

Volatiles may be also used for fast and reliable detection of pathogen infections as pathogens emit volatiles as well. For example, by analyzing the volatile profile of plant pathogenic fungi and oomycetes, we revealed that each isolate emits a specific blend of volatiles [24]. Another study investigated the breath volatile profile of swine infected with Influenza A, resulting in volatiles that could be related to the infection [95]. Other studies worked with human cells lines (lung epithelium) and combined those with *Pseudomonas aeruginosa* causing ventilator-associated pneumonia showing likewise volatiles that could potentially be used as biomarkers [96,97]. *Staphylococcus aureus* is a common bacterium infecting children with cystic fibrosis and a recent study detected this pathogen in cystic fibrosis patients using breath volatile profiles [98].

In agriculture as well volatiles can provide information of early pathogen infractions. Volatiles emitted during the infection of apple plants by bacterial pathogens *Erwinia amylovora* or *Pseudomonas syringae* pv. *syringae* were studied by gas chromatography-mass spectrometry and proton transfer reaction-mass spectrometry. Infected plants showed a disease-specific emission of volatiles, including several bioactive compounds, such as hexenal isomers and 2,3-butanediol [99]. Those approaches are non-invasive, fast, reliable and have the potential to avoid the prophylactic and wrongly use of antibiotics.

Finally, the potential use of volatiles as antimicrobials is often criticized because of their physicochemical properties. In fact, numerous volatiles are liquid at room temperature if not solid and could therefore be solved in appropriate solvents. Furthermore, Avalos suggested the inhaling of volatiles as possible therapy in future [18]. We are convinced that innovative and novel approaches are needed, and antimicrobial volatiles could be a future solution.

## Figures and Tables

**Table 1:** Overview of recent (2017–2021) studies showing the antimicrobial activity of bacterial volatiles. The studies are ordered alphabetically by the volatile producer’s name. Only studies that trapped the antimicrobial volatiles in the gas phase and/or showed the antimicrobial effect via the gas phase are listed. blend = The volatile blend might be analyzed in the cited study but only the antimicrobial activity of the blend was tested. f = antifungal, b = antibacterial, o = anti-oomycete.

Volatile Producer	Volatile(s)	Bioactivity	Reference
*Bacillus amyloliquefaciens* CPA-8	blend 1,3-pentadiene thiophene acetoine	f	[47]
*Bacillus amyloliquefaciens* DA12	blend	f	[48]
*Bacillus amyloliquefaciens* FZB42	blend 1,2-benzisothiazol-3(2*H*)-one benzaldehyde other	b	[49]
*Bacillus amyloliquefaciens* L3 other	blend 2-heptanone 2-ethyl-1-hexanol 2-nonanone other	f	[50]
*Bacillus artrophaeus* LSSC22	blend 1,2-benzisothiazol-3(2*H*)-one other	b	[49]
*Bacillus cereus* CHP20	blend	o	[44]
*Bacillus megaterium* KU143	blend	f	[51]
*Bacillus pumilus* TM-R	blend	f	[52]
*Bacillus siamensis* LZ88	blend	f	[53]
*Bacillus* (diverse spp.)	blend	f	[54]
*Bacillus* (diverse spp.)	blend 2-undecanone benzothiazole 1,3-butadiene *N*,*N*-dimethyldodecylamine other	f	[55]
*Bacillus* sp. BO53	blend	b	[43]
*Bacillus* sp. D13	blend	b	[56]
*Bacillus* sp. TM-I-3	blend	f	[57]
*Bacillus subtilis* CHP14	blend	o	[44]
*Bacillus subtilis* FA26	blend benzaldehyde nonanal benzothiazole acetophenone	b	[58]
*Bacillus subtilis* M29	blend 1-butanol acetic acid butyl ester 1-heptylene-4-alcohol 3-methyl-3-hexanol other	f	[59]
*Bacillus velezensis* BUZ-14	blend diacetyl benzaldehyde isoamyl alcohol other	f	[41]
*Bacillus velezensis* G341	blend	f	[60]
*Bacillus velezensis* I3	blend	f	[41]
bacterial community	blend	f	[31]
*Cronobacter muytjensii* JZ38	blend	o	[61]
*Frigoribacterium endophyticum* CHP33	blend	o	[44]
*Microbacterium testaceum* KU313	blend	f	[51]
*Paenibacillus* sp. AD87	2,5-*bis*(1-methylethyl)-pyrazine	b, f	[21]
*Paenibacillus polymyxa* Sb3-1	blend	f	[42]
*Proteus mirabilis* 04	blend	b	[62]
*Pseudoalteromonas* sp. GA327	blend	b	[43]
*Pseudomonas chlororaphis* subsp. *aurantiaca* KNU17Pc1	blend	f	[63]
*Pseudomonas chlororaphis* subsp. *aureofaciens* SPS-41	blend 3-methyl-1-butanol phenylethyl alcohol 2-methyl-1-butanol other	f	[64]
*Pseudomonas protegens* AS15	blend	f	[51]
*Pseudomonas protegens* CHA0	dimethyl trisulfide 2-ethylhexanol ammonium hydroxide phenol acetophenone 1,3-diphenylpropane 3-phenylpropiophenone	f	[65]
*Pseudomonas putida* BP25	blend 2-ethyl-5-methylpyrazine	f	[66]
*Pseudomonas putida* BP25	2,5-dimethyl pyrazine 2-methyl pyrazine 2-ethyl-5-methyl pyrazine 2-ethyl-3,6-dimethyl pyrazine dimethyl trisulfide	b, f, o	[67]
*Pseudomonas stutzeri* E25	blend dimethyl disulfide	f	[68]
*Sphingobacterium multivorum* Bel3-4	blend	f	[54]
*Stenotrophomonas maltophilia* CR71	blend dimethyl disulfide	f	[68]
*Stenotrophomonas maltophilia * (TD1 and GH1-5)	blend	f	[54]
*Streptomyces alboflavus* TD-1	blend anisole dimethyl trisulfide β-pinene benzenamine 1,5-cyclooctadiene	f	[69]
*Streptomyces fimicarius* BWL-H1	phenylethyl alcohol ethyl phenylacetate methyl anthranilate α-copaene caryophyllene methyl salicylate 4-ethylphenol	o	[70]
*Streptomyces lavendulae* SPS-33	blend 2-methyl-1-butanol 3-methyl-1-butanol pyridine phenylethyl alcohol other	f	[71]
*Streptomyces* sp. MBT11	blend	b	[39]
*Streptomyces venezuelae* (ATCC 15439)	blend	b	[39]
*Streptomyces yanglinensis* 3–10	blend methyl 2-methylbutyrate 2-phenylethanol β-caryophyllene	f, o f f f	[72]
*Xenorhabdus szentirmaii* PAM 25	blend	f	[73]

**Table 2:** Examples of bacterial volatiles that were upregulated or downregulated in co-cultures. blend = The volatile blend might be analyzed in the cited study but only the antimicrobial activity of the blend was tested. f = antifungal, b = antibacterial, o = anti-oomycete, na = not analyzed.

Co-Culture	Volatile(s)	Bioactivity	Reference
*Burkholderia* sp. AD24 *Paenibacillus* sp. AD87	2,5-*bis*(1-methylethyl)-pyrazine	b, f	[21]
*Burkholderia* sp. AD024 *Paenibacillus* sp. AD087 *Dyella* sp. AD056 *Janthinobacterium* sp. AD080 *Pseudomonas* sp. AD021	2,5-*bis*(1-methylethyl)-pyrazine other	na	[82]
*Chryseobacterium* sp. AD48 *Tsukamurella* sp. AD106	blend dimethyl trisulfide other	b, f, o b	[75]
*Janthinobacterium* sp. AD80 *Dyella* sp. AD56	blend dimethyl trisulfide other	f, o b	[75]
*Paenibacillus polymyxa* Sb3-1 *Verticillium longisporum* EVL43	blend trans-2,2,4,5-tetramethyl-1,3-dioxolane 1-butanol other	f na na	[42]
*Serratia plymuthica* 4Rx13 *Staphylococcus delphini*	schleiferon A and B	na	[83]

## References

[1] Dodds D.R. (2017). Antibiotic Resistance: A Current Epilogue. Biochem. Pharmacol..

[2] Butler M.S., Buss A.D. (2006). Natural Products-The Future Scaffolds for Novel Antibiotics?. Biochem. Pharmacol..

[3] Rolain J.-M., Abat C., Jimeno M.-T., Fournier P.-E., Raoult D. (2016). Do We Need New Antibiotics?. Clin. Microbiol. Infect..

[4] Casewell M., Hill R. (1985). In-Vitro Activity of Mupirocin (‘Pseudomonic Acid’) against Clinical Isolates of *Staphylococcus aureus*. J. Antimicrob. Chemother..

[5] Sutherland R., Boon R., Griffin K., Masters P., Slocombe B., White A. (1985). Antibacterial Activity of Mupirocin (Pseudomonic Acid), a New Antibiotic for Topical Use. Antimicrob. Agents Chemother..

[6] Clemett D., Markham A. (2000). Linezolid. Drugs.

[7] LaPlante K.L., Rybak M.J. (2004). Daptomycin–a Novel Antibiotic against Gram-Positive Pathogens. Expert Opin. Pharmacother..

[8] Ling L.L., Schneider T., Peoples A.J., Spoering A.L., Engels I., Conlon B.P., Mueller A., Schäberle T.F., Hughes D.E., Epstein S. (2015). A New Antibiotic Kills Pathogens without Detectable Resistance. Nature.

[9] Fleming A. Penicillin. https://www.nobelprize.org/prizes/medicine/1945/fleming/lecture/.

[10] Michael C.A., Dominey-Howes D., Labbate M. (2014). The Antimicrobial Resistance Crisis: Causes, Consequences, and Management. Front. Public Health.

[11] WHO No Time to Wait: Securing the Future from Drug-Resistant Infections. https://www.who.int/publications/i/item/no-time-to-wait-securing-the-future-from-drug-resistant-infections.

[12] Hwang A.Y., Gums J.G. (2016). The Emergence and Evolution of Antimicrobial Resistance: Impact on a Global Scale. Bioorg. Med. Chem..

[13] O’Neill J. Tackling Drug-Resistant Infections Globally: Final Report and Recommendations. https://amr-review.org/Publications.html.

[14] Banin E., Hughes D., Kuipers O.P. (2017). Editorial: Bacterial Pathogens, Antibiotics and Antibiotic Resistance. FEMS Microbiol. Rev..

[15] Getahun H., Smith I., Trivedi K., Paulin S., Balkhy H.H. (2020). Tackling Antimicrobial Resistance in the COVID-19 Pandemic. Bull. World Health Organ..

[16] Czaplewski L., Bax R., Clokie M., Dawson M., Fairhead H., Fischetti V.A., Foster S., Gilmore B.F., Hancock R.E.W., Harper D. (2016). Alternatives to Antibiotics—A Pipeline Portfolio Review. Lancet Infect. Dis..

[17] Coates A.R., Halls G., Hu Y. (2011). Novel Classes of Antibiotics or More of the Same?. Br. J. Pharmacol..

[18] Avalos M., van Wezel G.P., Raaijmakers J.M., Garbeva P. (2018). Healthy Scents: Microbial Volatiles as New Frontier in Antibiotic Research?. Curr. Opin. Microbiol..

[19] Mohamed H., Hassane A., Rawway M., El-Sayed M., Gomaa A.E.-R., Abdul-Raouf U., Shah A.M., Abdelmotaal H., Song Y. (2021). Antibacterial and Cytotoxic Potency of Thermophilic *Streptomyces Werraensis* MI-S.24-3 Isolated from an Egyptian Extreme Environment. Arch. Microbiol..

[20] Nas F., Aissaoui N., Mahjoubi M., Mosbah A., Arab M., Abdelwahed S., Khrouf R., Masmoudi A.-S., Cherif A., Klouche-Khelil N. (2021). A Comparative GC-MS Analysis of Bioactive Secondary Metabolites Produced by Halotolerant *Bacillus* spp. Isolated from the Great Sebkha of Oran. Int. Microbiol..

[21] Tyc O., de Jager V.C.L., van den Berg M., Gerards S., Janssens T.K.S., Zaagman N., Kai M., Svatos A., Zweers H., Hordijk C. (2017). Exploring Bacterial Interspecific Interactions for Discovery of Novel Antimicrobial Compounds. Microb. Biotechnol..

[22] Zhao B., Lin X., Lei L., Lamb D.C., Kelly S.L., Waterman M.R., Cane D.E. (2008). Biosynthesis of the Sesquiterpene Antibiotic Albaflavenone in *Streptomyces Coelicolor* A3(2). J. Biol. Chem..

[23] Rowan D.D. (2011). Volatile Metabolites. Metabolites.

[24] Schmidt R., Cordovez V., de Boer W., Raaijmakers J., Garbeva P. (2015). Volatile Affairs in Microbial Interactions. ISME J..

[25] Schulz-Bohm K., Martin-Sanchez L., Garbeva P. (2017). Microbial Volatiles: Small Molecules with an Important Role in Intra- and Inter-Kingdom Interactions. Front. Microbiol..

[26] Weisskopf L., Schulz S., Garbeva P. (2021). Microbial Volatile Organic Compounds in Intra-Kingdom and Inter-Kingdom Interactions. Nat. Rev. Microbiol..

[27] Ossowicki A., Jafra S., Garbeva P. (2017). The Antimicrobial Volatile Power of the Rhizospheric Isolate *Pseudomonas donghuensis* P482. PLoS ONE.

[28] Chen J., Henderson G., Grimm C.C., Lloyd S.W., Laine R.A. (1998). Termites Fumigate Their Nests with Naphthalene. Nature.

[29] Lammers A., Zweers H., Sandfeld T., Bilde T., Garbeva P., Schramm A., Lalk M. (2021). Antimicrobial Compounds in the Volatilome of Social Spider Communities. Front. Microbiol..

[30] Garbeva P., Hordijk C., Gerards S., de Boer W. (2014). Volatile-Mediated Interactions between Phylogenetically Different Soil Bacteria. Front. Microbiol..

[31] Li X., Garbeva P., Liu X., Gunnewiek P.J.A.K., Clocchiatti A., Hundscheid M.P.J., Wang X., de Boer W. (2020). Volatile-Mediated Antagonism of Soil Bacterial Communities against Fungi. Environ. Microbiol..

[32] Schulz S., Dickschat J.S. (2007). Bacterial Volatiles: The Smell of Small Organisms. Nat. Prod. Rep..

[33] Peñuelas J., Asensio D., Tholl D., Wenke K., Rosenkranz M., Piechulla B., Schnitzler J.p. (2014). Biogenic Volatile Emissions from the Soil. Plant Cell Environ..

[34] Ryu C.M., Farag M.A., Hu C.H., Reddy M.S., Wei H.X., Pare P.W., Kloepper J.W. (2003). Bacterial Volatiles Promote Growth in *Arabidopsis*. Proc. Natl. Acad. Sci. USA.

[35] Hammerbacher A., Coutinho T.A., Gershenzon J. (2019). Roles of Plant Volatiles in Defence against Microbial Pathogens and Microbial Exploitation of Volatiles. Plant Cell Environ..

[36] Veselova M.A., Plyuta V.A., Khmel I.A. (2019). Volatile Compounds of Bacterial Origin: Structure, Biosynthesis, and Biological Activity. Microbiology.

[37] Buhaescu I., Izzedine H. (2007). Mevalonate Pathway: A Review of Clinical and Therapeutical Implications. Clin. Biochem..

[38] Rohdich F., Zepeck F., Adam P., Hecht S., Kaiser J., Laupitz R., Gräwert T., Amslinger S., Eisenreich W., Bacher A. (2003). The Deoxyxylulose Phosphate Pathway of Isoprenoid Biosynthesis: Studies on the Mechanisms of the Reactions Catalyzed by IspG and IspH Protein. Proc. Natl. Acad. Sci. USA.

[39] Avalos M., Garbeva P., Raaijmakers J.M., van Wezel G.P. (2019). Production of Ammonia as a Low-Cost and Long-Distance Antibiotic Strategy by *Streptomyces* Species. ISME J..

[40] Bernier S.P., Letoffe S., Delepierre M., Ghigo J.-M. (2011). Biogenic Ammonia Modifies Antibiotic Resistance at a Distance in Physically Separated Bacteria. Mol. Microbiol..

[41] Calvo H., Mendiara I., Arias E., Gracia A.P., Blanco D., Eugenia Venturini M. (2020). Antifungal Activity of the Volatile Organic Compounds Produced by *Bacillus velezensis* Strains against Postharvest Fungal Pathogens. Postharvest Biol. Technol..

[42] Rybakova D., Rack-Wetzlinger U., Cernava T., Schaefer A., Schmuck M., Berg G. (2017). Aerial Warfare: A Volatile Dialogue between the Plant Pathogen *Verticillium longisporum* and Its Antagonist *Paenibacillus polymyxa*. Front. Plant Sci..

[43] Garrido A., Atencio L.A., Bethancourt R., Bethancourt A., Guzmán H., Gutiérrez M., Durant-Archibold A.A. (2020). Antibacterial Activity of Volatile Organic Compounds Produced by the Octocoral-Associated Bacteria *Bacillus* sp. BO53 and *Pseudoalteromonas* sp. GA327. Antibiotics.

[44] Bruisson S., Zufferey M., L’Haridon F., Trutmann E., Anand A., Dutartre A., De Vrieze M., Weisskopf L. (2019). Endophytes and Epiphytes From the Grapevine Leaf Microbiome as Potential Biocontrol Agents against Phytopathogens. Front. Microbiol..

[45] Janssens T.K.S., Tyc O., Besselink H., de Boer W., Garbeva P. (2019). Biological Activities Associated with the Volatile Compound 2,5-*bis*(1-Methylethyl)-pyrazine. Fems Microbiol. Lett..

[46] Schulz S., Dickschat J.S., Kunze B., Wagner-Dobler I., Diestel R., Sasse F. (2010). Biological Activity of Volatiles from Marine and Terrestrial Bacteria. Mar. Drugs.

[47] Gotor-Vila A., Teixidó N., Di Francesco A., Usall J., Ugolini L., Torres R., Mari M. (2017). Antifungal Effect of Volatile Organic Compounds Produced by *Bacillus amyloliquefaciens* CPA-8 against Fruit Pathogen Decays of Cherry. Food Microbiol..

[48] Lee T., Park D., Kim K., Lim S.M., Yu N.H., Kim S., Kim H.-Y., Jung K.S., Jang J.Y., Park J.-C. (2017). Characterization of *Bacillus amyloliquefaciens* DA12 Showing Potent Antifungal Activity against Mycotoxigenic *Fusarium* Species. Plant Pathol. J..

[49] Tahir H.A.S., Gu Q., Wu H., Niu Y., Huo R., Gao X. (2017). Bacillus Volatiles Adversely Affect the Physiology and Ultra-Structure of *Ralstonia Solanacearum* and Induce Systemic Resistance in Tobacco against Bacterial Wilt. Sci. Rep..

[50] Wu Y., Zhou J., Li C., Ma Y. (2019). Antifungal and Plant Growth Promotion Activity of Volatile Organic Compounds Produced by *Bacillus amyloliquefaciens*. MicrobiologyOpen.

[51] Mannaa M., Kim K.D. (2018). Biocontrol Activity of Volatile-Producing *Bacillus megaterium* and *Pseudomonas protegens* against *Aspergillus* and *Penicillium* spp. Predominant in Stored Rice Grains: Study II. Mycobiology.

[52] Morita T., Tanaka I., Ryuda N., Ikari M., Ueno D., Someya T. (2019). Antifungal Spectrum Characterization and Identification of Strong Volatile Organic Compounds Produced by *Bacillus pumilus* TM-R. Heliyon.

[53] Xie Z., Li M., Wang D., Wang F., Shen H., Sun G., Feng C., Wang X., Chen D., Sun X. (2021). Biocontrol Efficacy of *Bacillus siamensis* LZ88 against Brown Spot Disease of Tobacco Caused by *Alternaria alternata*. Biol. Control.

[54] Ezrari S., Mhidra O., Radouane N., Tahiri A., Polizzi G., Lazraq A., Lahlali R. (2021). Potential Role of Rhizobacteria Isolated from Citrus Rhizosphere for Biological Control of Citrus Dry Root Rot. Plants.

[55] Massawe V.C., Hanif A., Farzand A., Mburu D.K., Ochola S.O., Wu L., Tahir H.A.S., Gu Q., Wu H., Gao X. (2018). Volatile Compounds of Endophytic *Bacillus* spp. Have Biocontrol Activity against *Sclerotinia sclerotiorum*. Phytopathology.

[56] Xie S., Zang H., Wu H., Rajer F.U., Gao X. (2018). Antibacterial Effects of Volatiles Produced by *Bacillus* Strain D13 against *Xanthomonas Oryzae Pv. Oryzae*. Mol. Plant Pathol..

[57] Osaki C., Yamaguchi K., Urakawa S., Nakashima Y., Sugita K., Nagaishi M., Mitsuiki S., Kuraoka T., Ogawa Y., Sato H. (2019). The Bacteriological Properties of *Bacillus* Strain TM-I-3 and Analysis of the Volatile Antifungal Compounds Emitted by This Bacteria. Biocontrol Sci..

[58] Rajer F.U., Wu H., Xie Y., Xie S., Raza W., Tahir H.A.S., Gao X. (2017). Volatile Organic Compounds Produced by a Soil-Isolate, *Bacillus subtilis* FA26 Induce Adverse Ultra-Structural Changes to the Cells of *Clavibacter michiganensis* ssp. *sepedonicus*, the Causal Agent of Bacterial Ring Rot of Potato. Microbiology.

[59] Mu J., Li X., Jiao J., Ji G., Wu J., Hu F., Li H. (2017). Biocontrol Potential of Vermicompost through Antifungal Volatiles Produced by Indigenous Bacteria. Biol. Control.

[60] Lim S.M., Yoon M.-Y., Choi G.J., Choi Y.H., Jang K.S., Shin T.S., Park H.W., Yu N.H., Kim Y.H., Kim J.-C. (2017). Diffusible and Volatile Antifungal Compounds Produced by an Antagonistic *Bacillus velezensis* G341 against Various Phytopathogenic Fungi. Plant Pathol. J..

[61] Eida A.A., Bougouffa S., L’Haridon F., Alam I., Weisskopf L., Bajic V.B., Saad M.M., Hirt H. (2020). Genome Insights of the Plant-Growth Promoting Bacterium *Cronobacter muytjensii* JZ38 with Volatile-Mediated Antagonistic Activity against *Phytophthora infestans*. Front. Microbiol..

[62] Juarez G.E., Mateyca C., Galvan E.M. (2020). *Proteus mirabilis* Outcompetes *Klebsiella pneumoniae* in Artificial Urine Medium through Secretion of Ammonia and Other Volatile Compounds. Heliyon.

[63] Tagele S.B., Lee H.G., Kim S.W., Lee Y.S. (2019). Phenazine and 1-Undecene Producing *Pseudomonas Chlororaphis* Subsp. *Aurantiaca* Strain KNU17Pc1 for Growth Promotion and Disease Suppression in Korean Maize Cultivars. Environ. Microbiol. Biotechnol..

[64] Zhang Y., Li T., Liu Y., Li X., Zhang C., Feng Z., Peng X., Li Z., Qin S., Xing K. (2019). Volatile Organic Compounds Produced by *Pseudomonas chlororaphis* subsp. *aureofaciens* SPS-41 as Biological Fumigants to Control *Ceratocystis fimbriata* in Postharvest Sweet Potatoes. J. Agric. Food Chem..

[65] Prigigallo M.I., De Stradis A., Anand A., Mannerucci F., L’Haridon F., Weisskopf L., Bubici G. (2021). *Basidiomycetes* Are Particularly Sensitive to Bacterial Volatile Compounds: Mechanistic Insight Into the Case Study of *Pseudomonas protegens* Volatilome against *Heterobasidion abietinum*. Front. Microbiol..

[66] Archana T.J., Gogoi R., Kaur C., Varghese E., Sharma R.R., Srivastav M., Tomar M., Kumar M., Kumar A. (2021). Bacterial Volatile Mediated Suppression of Postharvest Anthracnose and Quality Enhancement in Mango. Postharvest Biol. Technol..

[67] Agisha V.N., Kumar A., Eapen S.J., Sheoran N., Suseelabhai R. (2019). Broad-Spectrum Antimicrobial Activity of Volatile Organic Compounds from Endophytic *Pseudomonas putida* BP25 against Diverse Plant Pathogens. Biocontrol Sci. Technol..

[68] Rojas-Solis D., Zetter-Salmon E., Contreras-Perez M., del Carmen Rocha-Granados M., Macias-Rodriguez L., Santoyo G. (2018). *Pseudomonas stutzeri* E25 and *Stenotrophomonas maltophilia* CR71 Endophytes Produce Antifungal Volatile Organic Compounds and Exhibit Additive Plant Growth-Promoting Effects. Biocatal. Agric. Biotechnol..

[69] Yang M., Lu L., Pang J., Hu Y., Guo Q., Li Z., Wu S., Liu H., Wang C. (2019). Biocontrol Activity of Volatile Organic Compounds from *Streptomyces alboflavus* TD-1 against *Aspergillus flavus* Growth and Aflatoxin Production. J. Microbiol..

[70] Xing M., Zheng L., Deng Y., Xu D., Xi P., Li M., Kong G., Jiang Z. (2018). Antifungal Activity of Natural Volatile Organic Compounds against Litchi Downy Blight Pathogen *Peronophythora litchii*. Molecules.

[71] Li X., Li B., Cai S., Zhang Y., Xu M., Zhang C., Yuan B., Xing K., Qin S. (2020). Identification of Rhizospheric Actinomycete *Streptomyces lavendulae* SPS-33 and the Inhibitory Effect of Its Volatile Organic Compounds against *Ceratocystis fimbriata* in Postharvest Sweet Potato (*Ipomoea batatas* (L.) Lam.). Microorganisms.

[72] Lyu A., Yang L., Wu M., Zhang J., Li G. (2020). High Efficacy of the Volatile Organic Compounds of *Streptomyces yanglinensis* 3-10 in Suppression of *Aspergillus* Contamination on Peanut Kernels. Front. Microbiol..

[73] Chacon-Orozco J.G., Bueno C.J., Shapiro-Ilan D.I., Hazir S., Leite L.G., Harakava R. (2020). Antifungal Activity of *Xenorhabdus* spp. and *Photorhabdus* spp. against the Soybean Pathogenic *Sclerotinia sclerotiorum*. Sci. Rep..

[74] Dandurishvili N., Toklikishvili N., Ovadis M., Eliashvili P., Giorgobiani N., Keshelava R., Tediashvili M., Vainstein A., Khmel I., Szegedi E. (2010). Broad-Range Antagonistic Rhizobacteria *Pseudomonas fluorescens* and *Serratia plymuthica* Suppress *Agrobacterium* Crown Gall Tumours on Tomato Plants. J. Appl. Microbiol..

[75] Tyc O., Zweers H., de Boer W., Garbeva P. (2015). Volatiles in Inter-Specific Bacterial Interactions. Front. Microbiol..

[76] Watve M.G., Tickoo R., Jog M.M., Bhole B.D. (2001). How Many Antibiotics Are Produced by the Genus *Streptomyces*?. Arch. Microbiol..

[77] Gürtler H., Pedersen R., Anthoni U., Christophersen C., Nielsen P.H., Wellington E.M., Pedersen C., Bock K. (1994). Albaflavenone, a Sesquiterpene Ketone with a Zizaene Skeleton Produced by a *Streptomycete* with a New Rope Morphology. J. Antibiot..

[78] Moody S.C., Zhao B., Lei L., Nelson D.R., Mullins J.G.L., Waterman M.R., Kelly S.L., Lamb D.C. (2012). Investigating Conservation of the Albaflavenone Biosynthetic Pathway and CYP170 Bifunctionality in *Streptomycetes*. FEBS J..

[79] Takamatsu S., Lin X., Nara A., Komatsu M., Cane D.E., Ikeda H. (2011). Characterization of a Silent Sesquiterpenoid Biosynthetic Pathway in *Streptomyces avermitilis* Controlling Epi-Isozizaene Albaflavenone Biosynthesis and Isolation of a New Oxidized Epi-Isozizaene Metabolite. Microb. Biotechnol..

[80] Braña A.F., Rodríguez M., Pahari P., Rohr J., García L.A., Blanco G. (2014). Activation and Silencing of Secondary Metabolites in *Streptomyces albus* and *Streptomyces lividans* after Transformation with Cosmids Containing the Thienamycin Gene Cluster from *Streptomyces cattleya*. Arch. Microbiol..

[81] Wu Y., Yuan J.E.Y., Raza W., Shen Q., Huang Q. (2015). Effects of Volatile Organic Compounds from *Streptomyces albulus* NJZJSA2 on Growth of Two Fungal Pathogens. J. Basic Microbiol..

[82] Schulz-Bohm K., Zweers H., de Boer W., Garbeva P. (2015). A Fragrant Neighborhood: Volatile Mediated Bacterial Interactions in Soil. Front. Microbiol..

[83] Kai M., Effmert U., Lemfack M.C., Piechulla B. (2018). Interspecific Formation of the Antimicrobial Volatile Schleiferon. Sci. Rep..

[84] Abis L., Loubet B., Ciuraru R., Lafouge F., Houot S., Nowak V., Tripied J., Dequiedt S., Maron P.A., Sadet-Bourgeteau S. (2020). Reduced Microbial Diversity Induces Larger Volatile Organic Compound Emissions from Soils. Sci. Rep..

[85] Song C., Schmidt R., de Jager V., Krzyzanowska D., Jongedijk E., Cankar K., Beekwilder J., van Veen A., de Boer W., van Veen J.A. (2015). Exploring the Genomic Traits of Fungus-Feeding Bacterial Genus *Collimonas*. BMC Genom..

[86] Chernin L., Toklikishvili N., Ovadis M., Kim S., Ben-Ari J., Khmel I., Vainstein A. (2011). Quorum-Sensing Quenching by Rhizobacterial Volatiles. Environ. Microbiol. Rep..

[87] Trombetta D., Castelli F., Sarpietro M.G., Venuti V., Cristani M., Daniele C., Saija A., Mazzanti G., Bisignano G. (2005). Mechanisms of Antibacterial Action of Three Monoterpenes. Antimicrob. Agents Chemother..

[88] Garbeva P., Weisskopf L. (2019). Airborne Medicine: Bacterial Volatiles and Their Influence on Plant Health. New Phytol..

[89] Garbeva P., Hordijk C., Gerards S., De Boer W. (2014). Volatiles Produced by the Mycophagous Soil Bacterium *Collimonas*. FEMS Microbiol. Ecol..

[90] Singh R.P. (2011). A Method for Screening of Volatile Antimicrobial Compounds. Bull. Environ. Contam. Toxicol..

[91] Brożyna M., Żywicka A., Fijałkowski K., Gorczyca D., Oleksy-Wawrzyniak M., Dydak K., Migdał P., Dudek B., Bartoszewicz M., Junka A. (2020). The Novel Quantitative Assay for Measuring the Antibiofilm Activity of Volatile Compounds (AntiBioVol). Appl. Sci..

[92] Bonev B., Hooper J., Parisot J. (2008). Principles of Assessing Bacterial Susceptibility to Antibiotics Using the Agar Diffusion Method. J. Antimicrob. Chemother..

[93] Balouiri M., Sadiki M., Ibnsouda S.K. (2016). Methods for in Vitro Evaluating Antimicrobial Activity: A Review. J. Pharm. Anal..

[94] Létoffé S., Audrain B., Bernier S.P., Delepierre M., Ghigo J.-M. (2014). Aerial Exposure to the Bacterial Volatile Compound Trimethylamine Modifies Antibiotic Resistance of Physically Separated Bacteria by Raising Culture Medium PH. mBio.

[95] Traxler S., Bischoff A.-C., Saß R., Trefz P., Gierschner P., Brock B., Schwaiger T., Karte C., Blohm U., Schröder C. (2018). VOC Breath Profile in Spontaneously Breathing Awake Swine during Influenza A Infection. Sci. Rep..

[96] Lawal O., Knobel H., Weda H., Nijsen T.M.E., Goodacre R., Fowler S.J. (2018). TD/GC-MS Analysis of Volatile Markers Emitted from Mono- and Co-Cultures of *Enterobacter Cloacae* and *Pseudomonas Aeruginosa* in Artificial Sputum. Metabolomics.

[97] Lawal O., Knobel H., Weda H., Bos L.D., Nijsen T.M.E., Goodacre R., Fowler S.J. (2018). Volatile Organic Compound Signature from Co-Culture of Lung Epithelial Cell Line with *Pseudomonas aeruginosa*. Analyst.

[98] Neerincx A.H., Geurts B.P., van Loon J., Tiemes V., Jansen J.J., Harren F.J.M., Kluijtmans L.A.J., Merkus P.J.F.M., Cristescu S.M., Buydens L.M.C. (2016). Detection of *Staphylococcus Aureus* in Cystic Fibrosis Patients Using Breath VOC Profiles. J. Breath Res..

[99] Cellini A., Buriani G., Rocchi L., Rondelli E., Savioli S., Rodriguez Estrada M.T., Cristescu S.M., Costa G., Spinelli F. (2018). Biological Relevance of Volatile Organic Compounds Emitted during the Pathogenic Interactions between Apple Plants and *Erwinia amylovora*. Mol. Plant Pathol..

